# Population assignment and local adaptation along an isolation‐by‐distance gradient in Pacific cod (*Gadus macrocephalus*)

**DOI:** 10.1111/eva.12639

**Published:** 2018-05-23

**Authors:** Daniel P. Drinan, Kristen M. Gruenthal, Michael F. Canino, Dayv Lowry, Mary C. Fisher, Lorenz Hauser

**Affiliations:** ^1^ School of Aquatic and Fishery Sciences University of Washington Seattle Washington; ^2^ NOAA Fisheries Alaska Fisheries Science Center Seattle Washington; ^3^ Washington Department of Fish and Wildlife Olympia Washington

**Keywords:** fisheries management, genetic stock identification, Pacific cod, RAD sequencing, stock structure

## Abstract

The discernment of populations as management units is a fundamental prerequisite for sustainable exploitation of species. A lack of clear stock boundaries complicates not only the identification of spatial management units, but also the assessment of mixed fisheries by population assignment and mixed stock analysis. Many marine species, such as Pacific cod, are characterized by isolation by distance, showing significant differentiation but no clear stock boundaries. Here, we used restriction‐site‐associated DNA (RAD) sequencing to investigate population structure and assess power to genetically assign Pacific cod to putative populations of origin. Samples were collected across the species range in the eastern Pacific Ocean, from the Salish Sea to the Aleutian Islands. A total of 6,425 putative biallelic single nucleotide polymorphisms were identified from 276 individuals. We found a strong isolation‐by‐distance signal along coastlines that mirrored previous microsatellite results and pronounced genetic differentiation between coastal samples and those from the inland waters of the Salish Sea, with no evidence for hybridization between these two populations. Individual assignment success based on two methods was high overall (≥84%) but decreased from south to north. Assignment to geographic location of origin also was successful, with average distance between capture and assignment location of 220 km. Outlier analyses identified more loci potentially under selection along the coast than between Salish Sea and coastal samples, suggesting more diverse adaptation to latitudinal environmental factors than inshore vs. offshore environments. Our results confirm previous observations of sharp genetic differentiation of the Salish Sea population and isolation by distance along the coast, but also highlight the feasibility of using modern genomic techniques to inform stock boundaries and fisheries management in a low *F*_ST_ marine species.

## 1 INTRODUCTION

The identification of essentially self‐recruiting populations and the estimation of dispersal rates have been the primary aim of population genetics and management for many years (Palsboll, Berube, & Allendorf, 2007). In terrestrial and freshwater systems, the expectation of sharp boundaries between genetically differentiated populations is often confirmed by genetic data, allowing unequivocal delimitation of management units (Palsboll et al., 2007). In marine species, on the other hand, with their large population sizes and comparatively high rates of gene flow, population boundaries are often subtle (Waples, 1998), and populations may be structured by limited dispersal distances rather than clear barriers to gene flow (Wright, Bishop, Matthee, & von der Heyden, 2015). The resulting isolation‐by‐distance (IBD) patterns (Rousset, 1997; Slatkin, 1993) are of limited use to fisheries managers, because statistical areas cannot be clearly defined (Spies, Spencer, & Punt, 2015), and arbitrary delineation of stock boundaries is unacceptable to stakeholders. Genetic markers under selection that are often detected by next‐generation sequencing approaches may provide better resolution than more traditional markers (Hauser & Carvalho, 2008) and may also allow for the assessment of dispersal and seasonal migration based on individual assignment to population of origin, a technique that is rarely used in low *F*_ST_ marine species.

Pacific cod (*Gadus macrocephalus*) in the northeast Pacific Ocean is such a species, where restricted dispersal distances result in a clear IBD signal (Cunningham, Canino, Spies, & Hauser, 2009), thus complicating the identification of clear management units. Only cod in the Salish Sea have been identified as a clearly differentiated and spatially segregated population (Canino, Spies, Cunningham, Hauser, & Grant, 2010; Cunningham et al., 2009), which together with the declining abundance of cod in that region led to the listing of Pacific cod in 2010 as a NOAA Species of Concern in the Salish Sea. Along the Aleutian Islands, Gulf of Alaska, British Columbia, and Washington State, there was a tight IBD pattern without clear stock boundaries (Cunningham et al., 2009). A follow‐up study found an almost identical IBD pattern within Alaska but also identified subtle population boundaries (Spies, 2012), in part leading to the separation of the eastern Bering Sea and the Aleutian Islands management areas, which had been managed as a single unit until 2015. However, cod is still managed in three very large geographic management areas: eastern Bering Sea (EBS), Aleutian Islands (AI), and Gulf of Alaska (GOA), despite strong evidence for short generational dispersal distances <100 km (Cunningham et al., 2009) and consequent potential for population structure and local adaptation on much smaller geographic scales.

Pacific cod is an excellent system to test the power of next‐generation sequencing approaches to detect management units along an IBD gradient, not only because of the species’ limited distribution along the continental shelf, but also because of its significant economic, ecological, and conservation interest. Pacific cod is a demersal species distributed along the continental margin of the North Pacific to approximately 400‐m water depth (Cohen, Inada, Iwamoto, & Scialabba, 1990). There are two major phylogeographic groups in the northeastern and northwestern Pacific, which appear to represent recolonizations from different refugia with a boundary in the western Aleutians (Canino et al., 2010; Cunningham et al., 2009). In Alaska, Pacific cod move into shallower waters between 100‐m and 200‐m depth to aggregate for spawning in early spring (Neidetcher, Hurst, Ciannelli, & Logerwell, 2014). Spawning is widely distributed, with some concentrations associated with island topography (Neidetcher et al., 2014). Despite seasonal migrations, adult Pacific cod are noted for their spawning site fidelity and homing (Rand, Munro, Neidetcher, & Nichol, 2014; Shi, Gunderson, Munro, & Urban, 2007; Shimada & Kimura, 1994), which supports short lifetime dispersal estimates from genetic data (Cunningham et al., 2009). Fertilized Pacific cod eggs are negatively buoyant and adhesive until hatching (Fredlin, 1985), providing an additional mechanism to restrict dispersal distances. This distribution of spawning sites along a relatively narrow band along the shelf of the North American continent is expected to cause a linear IBD pattern (Slatkin, 1993), an expectation confirmed in two independent microsatellite studies (Cunningham et al., 2009; Spies, 2012).

Recent advances in next‐generation sequencing technologies have allowed for a more thorough survey of genetic variability and differentiation across the genome (Allendorf, Hohenlohe, & Luikart, 2010). One such technique, restriction‐site‐associated DNA (RAD) sequencing, has revolutionized population genetics in nonmodel organisms by allowing discovery and genotyping of thousands of single nucleotide polymorphisms (SNPs) in multiple individuals at relatively low cost (Baird et al., 2008; Miller, Dunham, Amores, Cresko, & Johnson, 2007). This large number of genetic markers not only affords much higher power in statistical tests of population differentiation and population assignment, but may also provide evidence for local adaptation from outlier loci that show particularly high differentiation among populations (Hauser & Seeb, 2008). Such outlier loci may be particularly valuable in discriminating populations where traditional markers show a simple IBD pattern without clear population boundaries (e.g., Atlantic cod in Norway, Hauser & Carvalho, 2008). Furthermore, outlier loci may provide valuable information on local adaptation of Pacific cod in the Salish Sea near the southern edge of the distribution, possibly explaining its continued decline despite protection for the past 30 years. In this study, we used RAD sequencing of Pacific cod to (i) increase resolution of stock boundaries along the coastal IBD gradient in the northeast Pacific Ocean from Washington State to the Aleutian Islands, (ii) evaluate the performance of methods to accurately assign individuals geographically to putative source populations, and (iii) address the potential for selective differentiation, both along the latitudinal gradient from Alaska to Washington and between Salish Sea cod and nearby coastal populations.

## 2 MATERIALS AND METHODS

### 2.1 Sample collection and RAD sequencing

**Figure 1:**
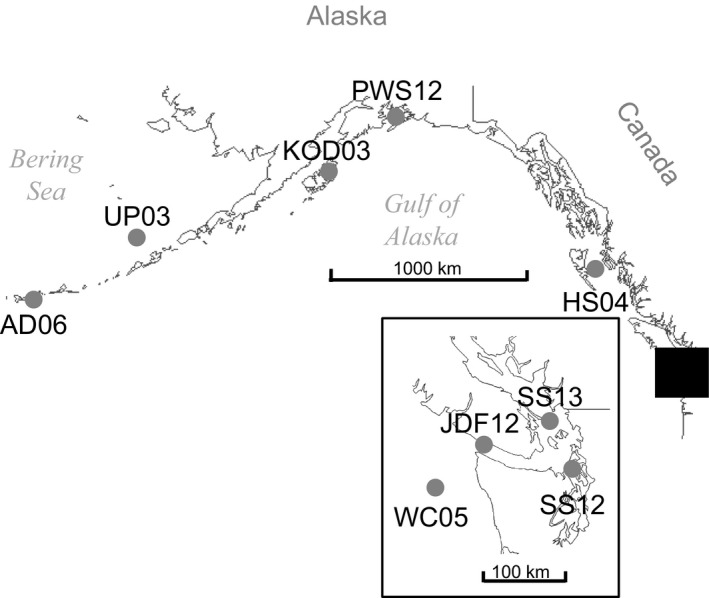
Map of northeastern Pacific collection sites for *Gadus macrocephalus*. Sample abbreviations are noted in Table 1. The black box denotes the position of the insert map

Samples were collected from spawning and prespawning aggregations of Pacific cod at seven locations across the northeastern Pacific Ocean (Table 1, Figure 1). An eighth sample of fish from the Salish Sea region was collected in 2012 in the United States and 2013 in Canadian waters (Figure 1 inset). Soft ray fin clips were preserved in 95%–100% nondenatured ethanol and stored at 4°C.

**Table 1:** Sample information for collection sites for *Gadus macrocephalus* in the northeastern Pacific

Location	Month/Year	Abbr.	MU	Lat.	Long.	*N*	Loci	*H*_*E*_	*N*_*e*_ (95% CI)
Salish Sea	May 2012	SS12	WA	48°14′N	122°40′W	17	5,845	0.19	2,861 (2,028, 4,850)
	August 2013	SS13	BC, 4B	49°34′N	124°31′W	10			
St. of Juan de Fuca	May 2012	JDF12	WA	48°8′N	122°40′W	17	5,629	0.19	3,479 (1,937, 16,913)
Washington Coast	February 2005	WC05	WA	47°55′N	125°33′W	38	6,317	0.19	2,326 (1,884, 3,038)
Hecate Strait	March 2004	HS04	BC, 5C & 5D	53°13′N	130°57′W	36	6,136	0.19	30,750 (7,852, Inf.)
Prince William Sound	March 2012	PWS12	GoA	60°32′N	147°4′W	45	6,267	0.20	8,648 (5,283, 23,751)
Kodiak Island	March 2003	KOD03	GoA	57°48′N	152°31′W	40	6,273	0.19	5,328 (3,639, 9,922)
Unimak Pass	January 2003	UP03	EBS	54°38′N	168°10′W	38	6,227	0.19	7,416 (4,446, 22,267)
Adak Island	March 2006	AD06	AI	51°40′N	176°36′W	35	6,313	0.19	2,832 (2,103, 4,326)

Data include population location name, month and year collected, abbreviation, management unit (MU: WA—Washington State; BC—British Columbia, Canada; GoA—Gulf of Alaska; EBS—eastern Bering Sea; AI—Aleutian Islands), approximate latitude and longitude, number of individuals (*n*), number of SNP loci out of 6425 possible, heterozygosity (*H*_*E*_), and genetically effective population size (*N*_*e*_).

DNA was extracted from fin clip tissue punches in 96‐well format using a DNeasy 96 Blood & Tissue Kit (Qiagen, Inc., Valencia, CA). RAD libraries were prepared, including *Sbf*I restriction enzyme digestion, adapter ligation, shearing, and PCR amplifications using 500 ng DNA per fish according to Baird et al. (2008) and Hohenlohe, Amish, Catchen, Allendorf, and Luikart (2011), with modification to include Agencourt AMPure XP SPRI beads (Beckman Coulter, Inc., Pasadena, CA) for size selection/exclusion and purification (P.D. Etter, University of Oregon, *personal communication*). Library sizes [300–1,000 base pair (bp) target length] were estimated with 1% E‐Gel EX agarose gels (Invitrogen, Carlsbad, CA). DNA concentrations and quality were assessed using Quant‐iT PicoGreen dsDNA Reagent (Invitrogen, Carlsbad, CA) and a FLx800 Fluorescence Microplate Reader (BioTek Instruments, Inc., Winooski, VT). Libraries were pooled within samples in 10 nM concentrations and sequenced in 100‐bp single‐end reads on a HiSeq2000 (Illumina, Inc., San Diego, CA) at the University of Oregon Genomics and Cell Characterization Core Facility (GC3F, Eugene, Oregon).

### 2.2 Data analysis

Raw data were converted to genotypes using Stacks v1.21 (Catchen, Hohenlohe, Bassham, Amores, & Cresko, 2013) according to the methods of Gruenthal et al. (2014), with minor modification. Briefly, catalogs created in the *cstacks* subprogram were generated from the five most data‐rich individuals from each sample. Flags (*m* = 3, *M* = 2, *N* = 4, *n* = 3, max_locus_stacks = 3) associated with increasing the number of loci, while reducing SNP and allele calling error rates, were set according to Mastretta‐Yanes et al. (2015). A genotype file containing putative polymorphic SNPs present in ≥80% of fish per sample was filtered to include one SNP per RAD tag (flag: write_random_SNP) to minimize physical linkage, and SNPs with more than two alleles were discarded. Final filtering removed the last nucleotide position on the tag (base pair 94) and loci with minor allele frequencies (MAFs) <0.05 to minimize sequencing errors. In addition, loci with uncorrected Hardy–Weinberg equilibrium (HWE) *p*‐values ≤.05 (global test) were removed to avoid inclusion of erroneous genotypes.

Locus‐specific allele frequencies, expected heterozygosity (*H*_E_), Hardy–Weinberg equilibrium (HWE) *p*‐values, and *F*‐statistics (*F*_IS_, *F*_ST_, and *F*_IT_) were estimated with GENEPOP v4.2 (Rousset, 2008) using the program default parameters. Population pairwise *F*_ST_ and associated *p*‐values were also estimated using GENEPOP v4.2. The genetically effective size (*N*_e_) of each population was estimated using NeEstimator v2.01 (Do et al., 2014) under the random mating model using the linkage disequilibrium method (Waples & Do, 2008), with an MAF cutoff of 0.05 (R. Waples, NOAA, *personal communication*).

Discriminant analysis of principal components (DAPC) as implemented within R (v3.3; R Core Team, 2017) using the package *adegenet* v1.4‐2 (Jombart, 2008; Jombart & Ahmed, 2011) was used to visualize relationships among individuals within samples. The number of principal components (PCs) retained in the analysis was determined using the function *optim.a.score*.

The presence and magnitude of global IBD were estimated with Mantel tests (999 permutations) run in GenAlEx v6.5b4 (Peakall & Smouse, 2012). To allow for comparison with previous microsatellite results (Cunningham et al., 2009), $G_{\mathrm{ST}}^{\prime \prime }$ (Meirmans & Hedrick, 2011) was estimated using *diveRsity* (Keenan, McGinnity, Cross, Crozier, & Prodöhl, 2013) within R for both microsatellite and RAD data, and pairwise linearized genetic distance ($G_{\mathrm{ST}}^{\prime \prime }$/(1‐$G_{\mathrm{ST}}^{\prime \prime }$)) was plotted against shortest geographic distance along the continental shelf estimated from Google Earth.

Coefficients of relatedness (*r*, percentage of alleles two individuals share by common descent) and putative genetic relationships (e.g., unrelated, siblingship, parent/offspring; 95% confidence level, 10^4^ simulations) between individuals were estimated and tested using the maximum‐likelihood framework in ML‐Relate (Kalinowski, Wagner, & Taper, 2006; Milligan, 2003).

### 2.3 Outlier testing and alignment to the Atlantic cod genome

Outlier tests to identify putative candidate loci under selection were performed using two different methods. First, a Bayesian framework with a differentiation‐based method was employed in BayeScan v2.1 (Foll & Gaggiotti, 2008). Testing was conducted with 20,000 iterations, prior odds of the neutral model of 100, and a type II false discovery rate of α = 0.05. Second, OutFLANK (Whitlock & Lotterhos, 2015) was used to identify candidate loci under selection. OutFLANK estimates the distribution of *F*_ST_ values at neutral, or nearly neutral, loci by fitting the empirical data to a chi‐square distribution after trimming excessively high and low *F*_ST_ values, as these loci may be under diversifying or balancing selection. The distribution is then compared to the empirical data, and outliers are identified as those outside the expected distribution. In this analysis, five percent of *F*_ST_ values were trimmed from each tail of the distribution before estimating the null chi‐square distribution. Samples from the coastal distribution in the northeastern Pacific (i.e., excluding the Salish Sea) were first analyzed to examine coastal patterns of selection, and then, a subset of samples (SS12/13, WC05, and HS04) were analyzed to examine differentiation associated with Salish Sea samples versus coastal samples at similar latitude.

To assess whether the outlier loci colocalized with regions identified as under selection in the congeneric Atlantic cod (*G. morhua*) by Hemmer‐Hansen, Therkildsen, Meldrup, and Nielsen (2014) and identify putative candidate genes involved in selection, Pacific cod sequences identified as outliers were aligned to the Atlantic cod genome (gadMor1 genome assembly, http://www.ensembl.org/Gadus_morhua) using BLASTN (Altschul, Gish, Miller, Myers, & Lipman, 1990). Scaffolds or contigs that aligned uniquely to each locus with an e‐value less than 10^−30^ were retained. Genome scaffolds were then assigned to linkage groups by alignment to an Atlantic cod linkage map (Borza, Higgins, Simpson, & Bowman, 2010) using Bowtie (Langmead, Trapnell, Pop, & Salzberg, 2009).

### 2.4 Population assignment tests

To assess the power of the data set to correctly assign individuals to their putative population of origin, we carried out three different assignment test procedures. First, individuals were assigned to populations in GeneClass2 (default settings; Piry et al., 2004) using a partial Bayesian method (Rannala & Mountain, 1997) and a leave‐one‐out procedure to avoid high‐grading bias (Anderson, 2010). All 6,425 loci were included in analyses, and results were compared to population assignment from microsatellite data (Cunningham et al., 2009).

Second, we tested our ability to assign individuals to their sample of origin with subsets of loci using Assigner (Gosselin, Anderson, & Bradbury, 2016). Assigner uses a training data set to identify highly discriminatory loci (based on *F*_ST_), followed by a leave‐one‐out method on an independent test data set to test the assignment (Anderson, 2010). Half the individuals in each sample were used as a training data set, and the remaining individuals were used to assign individuals to populations. Data sets with the 10, 50, 100, 200, 500, 1,000, and 6,425 (all) loci with the highest *F*_ST_ were used, with ten iterations per data set.

Third, we used an assignment test that assigns individuals to a geographic location within an IBD pattern rather than to a population of origin. SCAT (Smoothing and Continuous AssignmenTs, Wasser et al., 2004) assigned individuals relative to contiguous coastal samples (WC05, HS04, PWS12, KOD03, UP03, and AD06) in the northeastern Pacific using a leave‐one‐out approach. SCAT’s model is based on the assumption that allele frequencies vary in a continuous, predictable manner, as would be expected in IBD. SCAT uses a Bayesian framework to predict allele frequencies (with error) across the entirety of the geographic space. We used default model parameters (burn‐in = 100, iterations = 200, and MCMC steps per iteration = 10) and a geographic boundary box to limit the assignment of individuals to the continental shelf across the sampling region (Aleutian Islands to Washington coast). Each sample was assigned to a geographic location three times using different seed values to assess whether model parameters facilitated the convergence of results. The median value of the three assignments was used as the final assignment position. Correlations between assigned geographic locations of origin and capture locations were determined using a randomization test. Individuals were randomly assigned to capture locations, and the distance between the actual capture location and assigned geographic location of origin was compared between the randomized data set and the empirical data, with associated *p*‐values calculated in R (R Core Team, 2017).

### 2.5 Individual ancestry

Individual ancestry was estimated for all southeast samples (SS12/13, JDF12, WC05, and HS04) to further resolve population structure and gene flow between the Salish Sea and coastal samples. Individual ancestry was estimated using Structure (Pritchard, Stephens, & Donnelly, 2000). We used 50 000 and 100 000 burn‐in and MCMC iterations, respectively, and *K*‐values ranging from one to six run three times each. The optimal *K*‐value was determined using the method of Evanno, Regnaut, and Goudet (2005) implemented by Structure Harvester (Earl & Vonholdt, 2012). Individuals with a Q < 0.9 of any one population were considered of mixed ancestry (Vähä & Primmer, 2006). Similar analyses in coastal samples only reproduced the isolation‐by‐distance pattern shown by other analyses and are not shown.

## 3 RESULTS

A total of 6,425 putative biallelic SNP loci and 297 individuals were retained after filtering. Pairwise coefficients of relatedness (*r*) ranged from 0.00 to 0.99, with only 19 comparisons showing evidence for a genetic relationship other than unrelated (*r *=* *0.32 ± 0.251). Sixteen pairwise comparisons were at the half‐sibling level (*r *=* *0.22 ± .065) and three at full‐sibling status (*r *=* *0.84 ± 0.229; Table S1). Two putative full‐sibling pairs in SS12/13 generated *r* values in excess of 0.95, which was consistent with the same fish being sampled twice. The individual in each pair with fewer data was removed from further analyses. Of the remaining 17 comparisons, 13 individuals were in adjacent wells of the extraction and library preparation plates. Furthermore, at least one in each of 16 putative “sib” pairs had atypically high individual multilocus heterozygosity (*H*_I_ > 0.22), strongly suggesting contamination with adjacent sample wells. We therefore excluded all 17 individuals with a multilocus heterozygosity >0.22; half (*N* = 8) of these individuals were also one member of the contaminated pairs (Figure S1). The final data set thus comprised 276 individuals in total in all further analyses.

Locus‐specific allele frequencies, expected heterozygosity (*H*_E_), Hardy–Weinberg equilibrium (HWE) *p*‐values, locus‐specific *F*‐statistics (*F*_IS_, *F*_ST_, and *F*_IT_), and population pairwise *F*_ST_ values are reported in Table S2. Global *H*_E_ averaged 0.195 (*SD* = 0.0023) across populations (see also Table S2). Global *F*_IS_, *F*_ST_, and *F*_IT_ were 0.031, 0.015, and 0.045, respectively. Locus‐specific *F*_ST_ ranged from −0.018 to 0.714 and *F*_ST_ between sample pairs ranged from 0.001 to 0.043 (Table 2). All comparisons between samples were significant (*p *<* *0.001), except between the Strait of Juan de Fuca sample (JDF12) and the neighboring coast of Washington (WC05; *F*_ST_ = 0.0007, *p *=* *1.000) and Hecate Strait (HS04; *F*_*ST*_ = 0.0037, *p* = 1.000). The SS12/13 sample was the most divergent, and pairwise *F*_ST_ estimates with other samples ranged from 0.0148 with JDF12 to 0.0429 with AD06. Finally, *N*_e_ estimates averaged 7,955 individuals and ranged from 2,326 (95% CI of 1,884 to 3,038) for WC05 to 30,750 (95% CI of 7,852 to infinity) for HS04 (Table 1).

**Table 2:** Pairwise estimates of *F*_ST_ for *Gadus macrocephalus* in the northeastern Pacific

	SS12/13	JDF12	WC05	HS04	PWS12	KOD03	UP03
JDF12	**0.0148**						
WC05	**0.0187**	0.0007					
HS04	**0.0170**	0.0037	**0.0039**				
PWS12	**0.0303**	**0.0162**	**0.0159**	**0.0091**			
KOD03	**0.0354**	**0.0200**	**0.0189**	**0.0128**	**0.0030**		
UP03	**0.0353**	**0.0223**	**0.0204**	**0.0137**	**0.0031**	**0.0013**	
AD06	**0.0429**	**0.0287**	**0.0267**	**0.0189**	**0.0070**	**0.0044**	**0.0034**

All significant pairwise comparisons (*p* < 0.001) are in bold.

**Figure 2:**
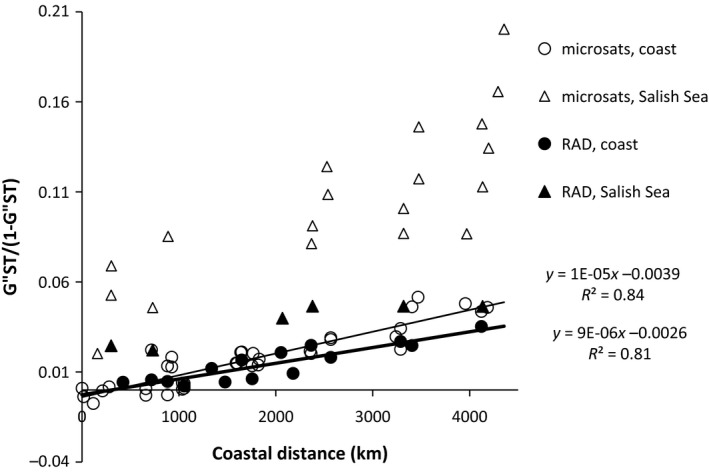
Global isolation by distance in *Gadus macrocephalus* from the northeastern Pacific from microsatellites (11 loci, open symbols) and RAD data (6,425 loci, full symbols). Triangles represent comparisons with samples from the Salish Sea

The IBD pattern along the coast was very similar to previous results with microsatellites (Figure 2). Slopes were almost identical between the two marker sets. However, samples from the Salish Sea were much more differentiated from coastal samples with microsatellites ($G_{\mathrm{ST}}^{\prime \prime }$ = 0.02–0.20) than with RAD sequences ($G_{\mathrm{ST}}^{\prime \prime }$ = 0.02‐0.05).

**Figure 3:**
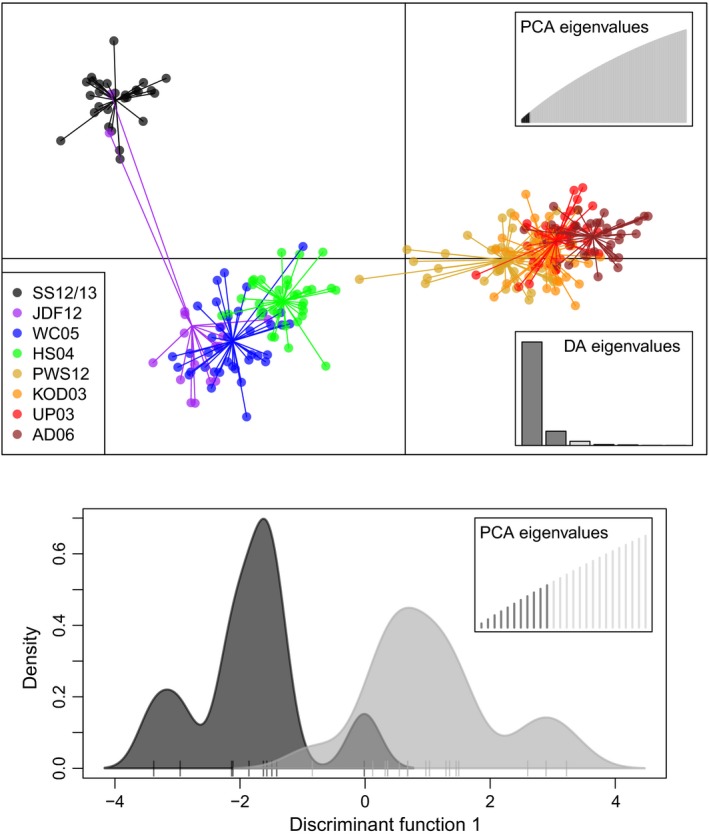
DAPC in *Gadus macrocephalus* from the northeastern Pacific, with all samples (top) and Salish Sea samples only (SS 12/13, bottom). Insets show number of PCs retained based on a‐score optimization. SS12/13 samples (bottom) are shown as a density chart of discriminant function 1, with northern Georgia Basin fish in dark gray and fish from U.S. waters (southern Georgia Basin, San Juan Island, and Puget Sound) in light gray. Hash marks along *x*‐axis (DF1) represent individual fish

Fourteen PCs were retained after a‐score optimization for DAPC on the full data set (Figure 3 top inset). Discriminant function 1 (DF1, *x*‐axis) largely reflected range‐wide IBD (Figure 3 top). The combination of DF1 and DF2 evidenced larger divisions among samples from the Salish Sea (SS12/13), southeastern coast (JDF12, WC05, and HS04), and northern range (PWS12, KOD03, UP03, and AD06). There was strong separation between SS12/13 and southeastern coastal samples (HS04 and WC04) despite small geographic distances (<500 km). A DAPC of Salish Sea samples revealed a possible division between samples collected in U.S. waters in 2012 and in Canadian waters (northern Georgia Basin) in 2013 (Figure 3 bottom).

### 3.1 Outlier testing and genome alignment

**Figure 4:**
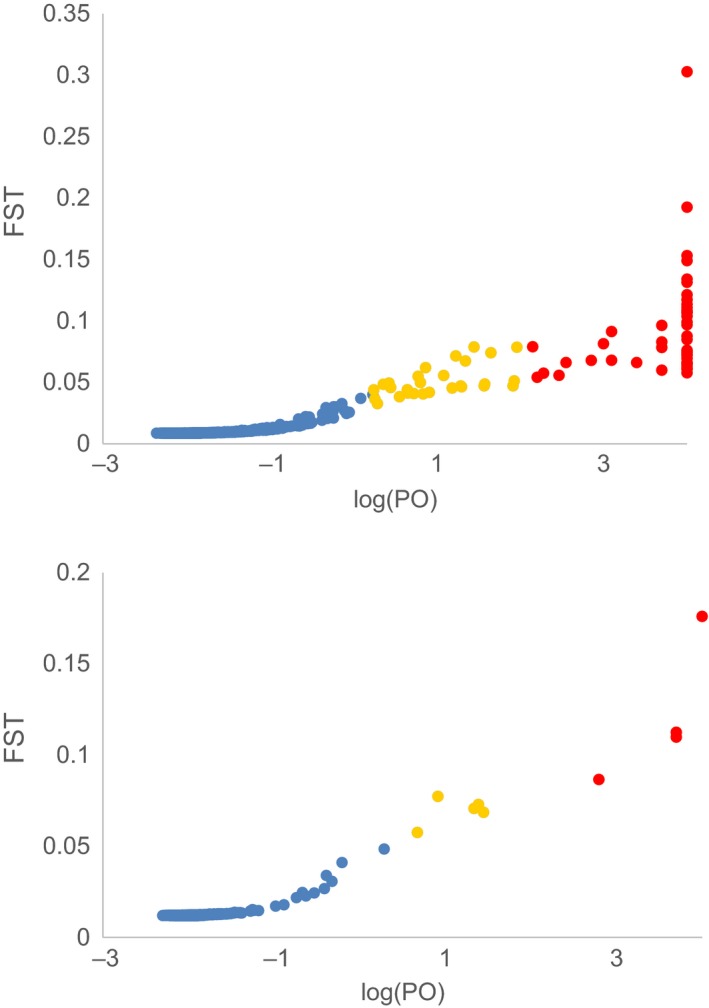
Outlier test results from BayeScan for *Gadus macrocephalus* from the northeastern Pacific. Per locus *F*_ST_ plotted against log10 probability of the odds (PO) for coastal samples in (a) and Salish Sea, WC05, and HS04 in (b). Loci with a *q* < 0.05 are shown in yellow and loci also with decisive evidence for selection (log(PO)>2) in red

Similar patterns of outlier loci were identified by both BayeScan and OutFLANK (Table 3). Across the coastal samples, 76 and 77 loci showed significant (FDR *q* < 0.05) evidence for selection by BayeScan (Figure 4a) and OutFLANK (Figure S2), respectively, and 65 loci were identified by both analyses (Table 3). BayeScan identified 48 of these loci as having “decisive” evidence for selection (log(PO)>2). Between coastal and Salish Sea samples (WC05 and HS04 vs. SS12/13), nine and 36 outlier loci were identified by BayeScan and OutFLANK, respectively, and seven loci were identified as outliers by both methods (Table 3). Four of the nine loci had decisive evidence for selection according to BayeScan (Figure 4b). None of the loci were identified as outliers in both comparisons by both methods (Table S3).

**Table 3:** Number of outliers (FDR *q* < 0.05) identified using BayeScan and OutFLANK on data sets containing just the coastal samples (AD06, UP03, KOD03, PWS12, HS04, and WC05) and another data set containing only the southeast samples (WC05 and HS04 vs SS12/13)

	BayeScan Coastal	BayeScan Southeast	OutFLANK Coastal	OutFLANK Southeast
BayeScanCoastal	76, 48 (54, 14)			
BayeScanSoutheast	2 (1, 0)	9, 4 (6, 1)		
OutFLANKCoastal	65 (44, 14)	3 (2, 0)	77 (54, 13)	
OutFLANKSoutheast	1 (1, 0)	7 (5, 1)	2 (2, 0)	36 (26, 2)

Numbers on the diagonal represent the total number of outliers identified by each test for each data set—for the two BayeScan analyses, the second number is the number of loci with decisive evidence for selection (log(PO)>2). Numbers below the diagonal represent the number of outliers in common between the two analyses. Numbers within parentheses represent how many outliers aligned uniquely to the Atlantic cod genome (gadMor1) and number of outliers that aligned uniquely to a coding region, respectively.

A large number of loci aligned uniquely to the Atlantic cod genome. Of the total 6,425 loci identified in this study, 5,007 aligned uniquely to the gadMor1 genome assembly and 866 of these aligned to coding regions. Forty‐four of 65 outlier loci identified by both BayeScan and OutFLANK in coastal samples aligned uniquely to the Atlantic cod genome and 14 aligned uniquely to coding regions (Tables 3 and S3). Five of seven outliers identified by both analyses in the comparison between the Salish Sea to coastal samples aligned to unique scaffolds and one aligned to a coding region (Tables 3 and S3). In total, four Atlantic cod scaffolds (GeneScaffold_1739, GeneScaffold_1755, GeneScaffold_1981, GeneScaffold_2531) had more than one outlier locus align to them in coastal comparisons, while one scaffold (GeneScaffold_2140) had more than one locus align in the Salish Sea versus coastal comparison. In total, 23 outlier loci were assigned to Atlantic cod linkage groups (Table S3). Four linkage groups (10, 14, 17, and 20) contained only one outlier, while linkage groups 1, 2, 8, 10, and 16 contained two, two, three, and four outlier loci, respectively (Table S3). Lastly, locus 15623 was the only outlier to colocalize to one of the same scaffolds (GeneScaffold_3590) as reported by Hemmer‐Hansen et al. (2014) for Atlantic cod.

### 3.2 Population assignment

Overall, GeneClass2 assigned 88% of individuals back to the sample from which they were captured (Table 4). This estimate excluded the Strait of Juan de Fuca because all individuals captured there were assigned either to the Washington Coast or to the Salish Sea. Assignment success declined latitudinally south to north, with almost perfect assignment in Washington State and BC and 77%–88% correct assignment in Alaska. This assignment success was considerably larger than the average achieved by microsatellites (32%) throughout the range—in fact, microsatellites only assigned twice as many individuals as expected by chance with six population samples (16%).

**Table 4:** Assignment results for *Gadus macrocephalus* populations from the northeastern Pacific

	SS12/13	JDF12	WC05	HS04	PWS12	KOD03	UP03	AD06	% Correct
SS12/13	27								100
JDF12	2		15						0
WC05			36	2					95
HS04			1	35					97
PWS12				1	37	4	3		82
KOD03					3	35	2		88
UP03					4	2	30	2	79
AD06					2		6	27	77

Numbers represent total individuals from samples in rows assigned to samples in columns. Percent correct assignment within samples is listed in column at right.

**Figure 5:**
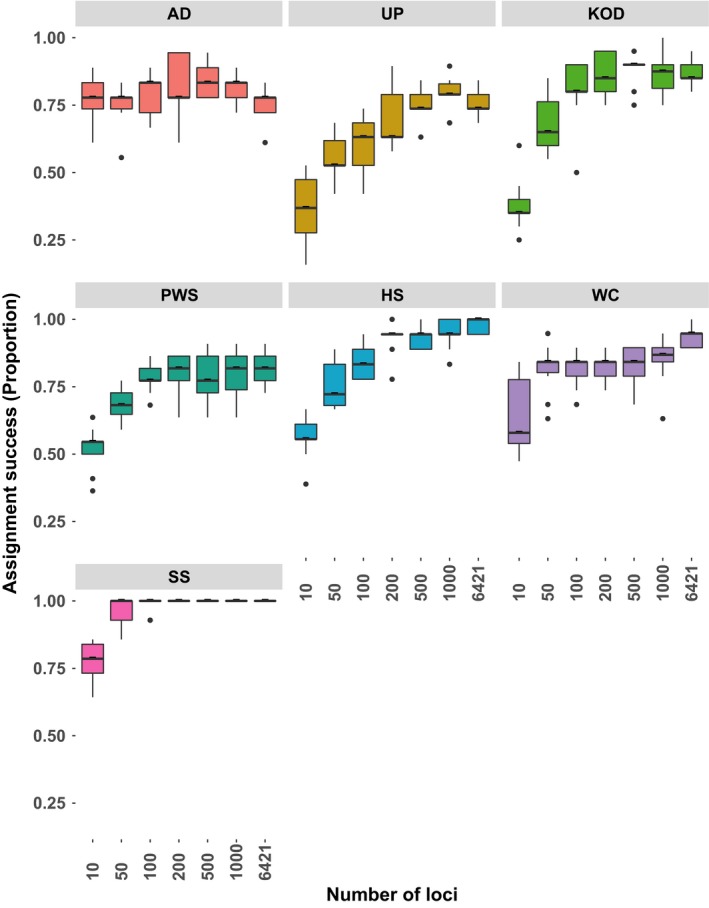
Assignment success of individuals to their sample of origin using the training, holdout, leave‐one‐out technique implemented by Assigner and GSI Sim. Number of loci used represents the highest *F*_*ST*_ markers identified in the training data set

Assignment success using Assigner depended both on the number of markers used (more was generally better) and the sample of origin (Figure 5). Median overall assignment accuracy was 61, 73, 78, 80, 82, 84, and 85% when using the top (based on *F*_ST_) 10, 50, 100, 200, 500, 1,000, and all loci, respectively. Some samples were easier to assign to their sample of origin than others. For example, 100% of individuals from the Salish Sea were correctly assigned to their sample of origin using only the top 50 markers. In contrast, only 78% of individuals from AD06 could be assigned to their sample of origin using all loci (Figures S3–S9). In addition, samples WC05 and AD06 both had little to no improvement in assignment accuracy by increasing the number of markers. In contrast, KOD03 had a nearly threefold increase in assignment accuracy (37.5% to 90%) by increasing from 10 to 200 loci. Not surprisingly, misassigned individuals were nearly always assigned to geographically neighboring samples (Figures S2–S8), likely due to the IBD pattern observed in Pacific cod.

**Figure 6:**
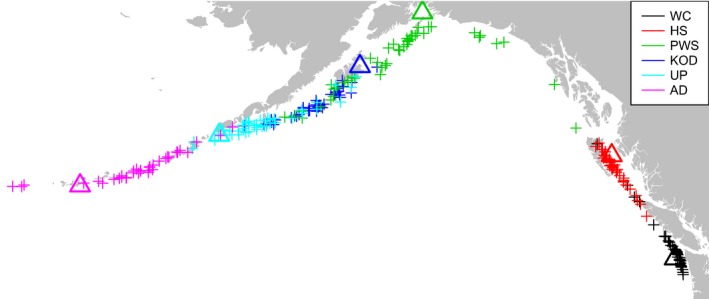
Assignment locations for each sample using the leave‐one‐out technique and SCAT to assign to a location of origin based on genetic background. Plus signs represent assignment locations, while triangles are capture locations

Assignments to geographic location of origin with the SCAT analyses were highly consistent among runs. The median distance between assigned geographic locations of origin in three replicates was 47 km per individual (25% and 75% quantiles = 28 and 74 km, respectively). Assignments for HS04 samples had the greatest precision, with a median difference among runs of 27 km (25% and 75% quantiles = 18 and 45 km), while AD06 had the greatest distance among runs with a median difference of 84 km (25% and 75% quantiles = 44 and 137 km, respectively). Assigned geographic locations of origin (median of three runs per sample) were highly correlated with capture locations (*p* < 0.001; Figures 6 and S10). The median distance between capture location and geographic assignment was 220 km (25% and 75% quantiles = 98 and 405 km) for all samples. In general, samples collected in the southeastern part of the range were assigned closer to their capture locations, with median distances between capture and assigned locations being 57 km (25% and 75% quantiles = 38 and 105 km) and 75 km (25 and 75% quantiles  53 and 150 km) for WC05 and HS04, respectively. In contrast, all other samples had an average distance between assigned geographic location of origin and capture location of 319 km (25 and 75% quantiles = 191 and 481). Among northern samples, assignments appeared to be pulled toward a center point between KOD03 and UP03. Likewise, samples from the south appeared to be drawn to a center point between HS04 and WC05 (Figure 6).

### 3.3 Individual ancestry

**Figure 7:**
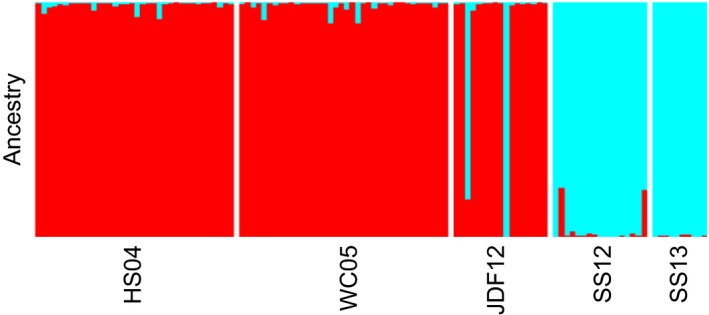
Ancestry results for samples from the southeastern portion of the range. Ancestry proportions were determined using Structure with 50,000 and 100,000 burn‐in and MCMC iterations and the previously determined optimal *K*‐value of 2

Structure results, with an optimal *K* value of two, suggested that JDF12 individuals were largely fish of coastal origin (Figure 7). All WC05 and HS04 fish clearly clustered together as a genetically homogenous group, while all SS13 and SS12 individuals clustered together as a distinct group in the Salish Sea. Of the JDF12 fish, 14 (87.5%) individuals had similar ancestries as coastal fish, and two JDF12 individuals appeared to cluster with the Salish Sea sample. Only three fish (2.6% of total; one JDF12 and two SS12) may have had mixed ancestries between the coast and Salish Sea (*Q* ≥ 0.10; Vähä & Primmer, 2006).

## 4 DISCUSSION

Our results confirmed the geographic patterns in population structure using other genetic markers. The IBD gradient previously reported for North American coastal Pacific cod using mitochondrial DNA sequences (Canino et al., 2010) and microsatellites (Cunningham et al., 2009; Spies, 2012) was here reproduced with SNPs. In addition, the relatively high genetic differentiation between the Salish Sea and coastal Pacific cod reported for microsatellites (Cunningham et al., 2009) and mtDNA (Canino et al., 2010) was supported by this study, although the level of differentiation was lower than in these previous studies. Despite this similarity in overall genetic patterns between the three marker systems, only RAD data provided the power to accurately assign individuals to population and location of origin.

### 4.1 Patterns of population differentiation

The slope of the IBD relationship was remarkably consistent with that found in two previous microsatellite studies (Cunningham et al., 2009; Spies, 2012), once estimates of differentiation were adjusted for differing levels of variability between marker systems (Meirmans & Hedrick, 2011). The two microsatellite studies used different microsatellite loci and different samples from a different part of the species range, so their consistency was already noteworthy, especially as overall *F*_ST_ values were tiny (max *F*_ST_ = 0.006, 0.003; Cunningham et al., 2009; Spies, 2012). The biological significance of such small *F*_ST_ estimates has been questioned because nonrandom sampling, scoring errors, or temporal fluctuations in allele frequencies may result in spurious but significant levels of differentiation (Knutsen et al., 2010; Waples, 1998). However, the confirmation of the slope by a completely different marker system further supports the genetic pattern itself and the underlying basis of limited dispersal distances, despite a lengthy larval period and highly migratory adults. It also supports the increasingly acknowledged pattern of subtle population structure in species with no obvious barriers to dispersal, such as marine fishes (Hauser & Carvalho, 2008).

The tight isolation‐by‐distance pattern along the coast seems to be a contradiction to the DAPC that very clearly shows three clusters, with two clusters along the coast (Figure 3). Sampling was not evenly spaced (Figure 1) as the commercial fishery is concentrated west of Prince William Sound (Barbeaux et al., 2017), and samples from southeast Alaska were not available. The clustering in the DAPC is therefore most likely to be a result of the geographic distribution of samples, rather than true genetic differentiation among isolated populations. The high correlation coefficient of the IBD relationship (*r* = 0.9, Figure 2) supports such an interpretation, and the DAPC clearly indicates IBD within Alaska (Figure 3), which was also found in an independent study (Spies, 2012). Furthermore, the previous microsatellite study failed to identify genetic barriers in that region (Cunningham et al., 2009). Nevertheless, finer scale sampling would be required to determine the exact mechanisms of genetic differentiation, which could either be caused by limited dispersal within a continuous population or a stepping‐stone pattern of migration along a series of isolated subpopulations (Meirmans, 2012).

The significant genetic differentiation documented previously between the Salish Sea and coastal Pacific cod (Canino et al., 2010; Cunningham et al., 2009) was also observed in this study. However, $G_{\mathrm{ST}}^{\prime \prime }$ between the two groups was much higher with microsatellites than with RAD tags (Figure 2). This difference was not due to selection at microsatellites. Eight of the 11 microsatellites supported the split between the two populations at roughly equal levels (Cunningham et al., 2009). Similarly, evidence for selective differentiation was found at only a few SNP loci and would result in the opposite pattern. However, microsatellite *R*_ST_ (an *F*_ST_ measure accounting for differences in allele sizes) was almost twice as high as *F*_ST_, suggesting mutation rather than just genetic drift as an important contributor to the observed differentiation (Canino et al., 2010). Mutations accumulate much faster in microsatellites than SNPs (Weber & Wong, 1993) and in isolated populations would arguably also have a larger effect in multiallelic microsatellites than in biallelic SNPs, where mutations do not lead to new alleles but only marginally change allele frequencies.

However, despite the lower levels of differentiation observed from RAD‐seq data in this study, ancestry estimates from Structure showed clearer separation between coastal and Salish Sea samples than for microsatellites (Canino et al., 2010), likely because of higher power conferred by the large number of loci. Indeed, there was little evidence for hybridization between the two populations (Figure 3), despite spatial overlap in the Strait of Juan de Fuca. Pacific cod collected in the Strait of Juan de Fuca did not constitute a “population,” but were instead made up mostly of individuals from the Washington coast and two Salish Sea cod, as indicated by GeneClass2, Structure, and DAPC results. Other than these two individuals, there was a sharp genetic break between the two populations near Victoria, BC, Canada. Similar genetic differentiation between Salish Sea and coastal populations has been found previously in hake (Iwamoto et al., 2015), brown rockfish (Buonaccorsi, Kimbrell, Lynn, & Vetter, 2005), and copper rockfish (Buonaccorsi, Kimbrell, Lynn, & Vetter, 2002).

This high differentiation with apparent lack of hybridization may seem surprising on such a small geographic scale and in an oceanographic system as dynamic as the Salish Sea. The genetic break coincides with the Victoria Sill, a shallow submarine feature that divides the Strait of Juan de Fuca from the interior Salish Sea (Ebbesmeyer & Barnes, 1980). This sill rises to depths of 55 m to 100 m and causes considerable vertical mixing during strong tidal currents in the Strait of Juan de Fuca (Ebbesmeyer & Barnes, 1980; Khangaonkar, Long, & Xu, 2017; Soontiens & Allen, 2017). This mixing creates spatial discontinuities in sediment load (Johannessen, Masson, & Macdonald, 2006), salinity (Masson & Cummins, 2000), nutrient load (Peña, Masson, & Callendar, 2016), and primary production (Masson & Peña, 2009). Sills are therefore seen as a mechanism limiting larval dispersal, and indeed, similar sills in Norwegian fjords seem to represent a barrier for the export of pelagic eggs of Atlantic cod, thus explaining the small‐scale differentiation found there (Knutsen et al., 2007). Adults, however, seem to be able to transverse such barriers, as evidenced by the two Salish Sea individuals found in the western Strait of Juan de Fuca. It therefore seems possible, that in addition to such extrinsic mechanisms maintaining isolation, intrinsic genomic mechanisms may be at play. Indeed, chromosome inversions in Atlantic cod in Scandinavia seem to enhance adaptive differentiation between populations (Barth et al., 2017), including stationary and migratory ecotypes (Berg et al., 2016), and populations inhabiting fjords and outer coasts (Sodeland et al., 2016). Such inversions are likely to cause genome regions of high differentiation among populations (“islands of divergence,” Sodeland et al., 2016). Given the weak evidence for adaptive divergence in Salish Sea cod found in our study, there is, however, currently no evidence for such inversions in Pacific cod.

A potential secondary split was found within the SS12/13 sample (Figure 3 bottom). Although more similar to one another than to any other sample (Figure 3 top), Pacific cod collected in 2012 in U.S. waters may be different from those collected in 2013 in the northern Georgia Basin in Canada. Consistent with the north–south break, another shallow sill is located north of the San Juan Islands at Boundary Pass (Masson, 2006), and the Fraser River freshwater plume and deltaic plain extends from Vancouver, BC, Canada, across the Basin toward Vancouver Island. One or both of these physical features may help isolate Pacific cod in the deeper northern Georgia Basin from those to the south. Indeed, freshwater and sediment barriers have been implicated in driving population divergence and endemism in marine taxa elsewhere. The Amazon, Yangtze, and Mississippi River outflows, for example, limit cross‐barrier dispersal in corals, limpets (*Cellana toreuma*), seahorses (*Hippocampus* spp.), various reef fishes, red snapper (*Lutjanus campechanus*), and other organisms (Boehm et al., 2013; Dong et al., 2012; Potts, 1983). Yet, it must be noted that temporal structuring, which was not examined in this study, cannot be ruled out.

The seemingly limited gene flow between Pacific cod in the Salish Sea and coastal populations may have important management implications. Pacific cod abundance in the Salish Sea has declined dramatically over recent decades (Johannessen & McCarter, 2010; Palsson, Hoeman, Bargmann, & Day, 1996). In 2000, the National Marine Fisheries Service published a status review of Pacific cod in Puget Sound and determined that their listing under the Endangered Species Act was not appropriate at the time (Gustafson et al., 2000). This determination was partially based on the lack of genetic information and an unclear northern boundary delineation that they hypothesized likely extends as far north as the border between British Columbia and southeastern Alaska. Microsatellite (Cunningham et al., 2009) and mtDNA (Canino et al., 2010) results already provided evidence for the genetic distinctness of Puget Sound cod, although the northern boundary of that population could still only be determined as south of Hecate Strait. The results presented here show that cod in the entire Salish Sea are differentiated from coastal populations, with very little evidence for ongoing gene flow and supporting a restrictive population delineation of Salish Sea cod including the Strait of Georgia, Puget Sound, and the eastern Strait of Juan de Fuca (Boundary 1 from Gustafson et al., 2000). Given these results, another status review of Salish Sea cod may be warranted.

### 4.2 Evidence for selective differentiation

There was strong evidence from outlier locus testing for selective differentiation across the range. Sixty‐five loci were putatively categorized as being under selection across the sampled coastal range in both outlier tests (BayeScan and OutFLANK), and seven were identified among HS04, WC05, and SS12/13. The identification of these loci by two different approaches supports the notion that they are truly under selection, although some of the observed patterns may still be the result of IBD (Lotterhos & Whitlock, 2014; Meirmans, 2012). The adaptive significance of these outlier loci is currently unknown, but candidate outlier genes identified by alignment to the Atlantic cod genome provide suggestive evidence that reproductive biology may play an important role in differentiation among coastal samples. First, locus 53780 had the largest overall *F*_ST_ value of any locus (*F*_ST_ = 0.71) and was identified as an outlier by both BayeScan and OutFLANK. This locus aligned to the coding region of a novel protein sequence that is orthologous to genes involved in reproduction and sexual development: steroidogenic factor 1 (SF1) and zona pellucida glycoprotein 3 (ZP3). Second, locus 8253 had the second largest overall *F*_ST_ value (*F*_ST_ = 0.46), was identified as an outlier in both coastal analyses, and aligned to an intron of the folliculogenesis‐specific bHLH transcription factor, which may regulate the expression of the reproductively important zona pellucida genes (Liang, Soyal, & Dean, 1997). Zona pellucida has been previously identified to undergo rapid selection in other species (Aagaard, Yi, MacCoss, & Swanson, 2006; Heras, McClintock, Sunagawa, & Aguilar, 2015), and both changes to coding regions as well as differences in expression patterns may be important to diversification in Pacific cod.

Fewer outlier loci were identified in the comparison between Salish Sea and coastal cod, a surprising result, given the sharp genetic break between populations and the dearth of identifiable hybrids. However, outlier tests have notoriously low power with few sampled populations (Foll & Gaggiotti, 2008), and the limited number of populations may have led to many false negatives. In addition, the overall high *F*_ST_ between coastal Pacific cod and Salish Sea cod may have reduced our power to detect outliers in that comparison. Nevertheless the strong genetic differentiation of Puget Sound cod (Cunningham et al., 2009) and their long isolation from coastal populations (Canino et al., 2010) provide the opportunity for adaptive genetic differentiation and local adaptation to warmer conditions in the southern limit of the distribution. Bottom temperatures during the larval period (February, March, 7–8°C; Moore et al., 2008) routinely exceed optimal temperatures for cod larval rearing (4°C; Alderdice & Forrester, 1971), suggesting potential effects of long‐term heat stress on growing larvae in Puget Sound. Similar patterns have been shown in Atlantic cod, where recruitment success of several stocks is tightly linked to sea surface temperatures during the larval period (Brander, 2010). Further evidence for local adaptation in Pacific cod stems from steep latitudinal clines of increased growth and mortality rates of Puget Sound cod (Karp, 1982), although the underlying genetic basis of such differences is currently unknown. Local adaptation has been demonstrated in Atlantic cod at very small geographic scales from both common garden experiments (Hutchings et al., 2007) and molecular studies (Case, Hutchinson, Hauser, Van Oosterhout, & Carvalho, 2005). Local adaptation increases the biocomplexity of the species as a whole, which may help prevent extreme abundance fluctuations in a portfolio effect (Schindler et al., 2010). Fisheries management approaches that incorporate evolutionary and plastic biocomplexity have been suggested for Atlantic cod, for example, in Olsen et al. (2008). Moreover, populations at the southern edge of the species’ distribution may be old and harbor genetic variation important for the survival and evolution of a species (i.e., rear edge effect; Hampe & Petit, 2005). These stabilizing characteristics may become increasingly important as climate change progresses (Hauser & Carvalho, 2008).

### 4.3 Population assignment

Poor population assignment rates are often expected for species exhibiting IBD (Pritchard et al., 2000) depending on the level of differentiation and the spatial scale of sampling. Nevertheless, RAD sequencing increased our ability to re‐assign individuals to their population of origin relative to microsatellite loci used in previous studies. Excluding JDF12, median reassignment success using all RAD tags was ≥85% for each method (Table 4 and Supporting information), while rates for microsatellites using GeneClass2 averaged only about 32% (L. Hauser, *unpublished data*). The higher assignment success of RAD sequencing is likely due to both the greater number of loci in this study and the inclusion of outlier loci with high levels of differentiation.

Despite the recent and ongoing decline in sequencing costs, RAD sequencing is too costly for routine application on large‐scale fisheries samples. However, simulations based on loci identified in a training data set and applied in a leave‐one‐out fashion on a holdout data set (Anderson, 2010) suggested that 100 to 200 loci may be sufficient to approach a similar assignment success as for all 6,425 loci. Such a set of 100 loci could be easily scored using reduced representation libraries (e.g., GT_SEQ_; Campbell, Harmon, & Narum, 2015), and opens potential applications to source verify catch or fish products (Nielsen et al., 2012), estimate contributions to mixed stock fisheries (Ruzzante, Taggart, Lang, & Cook, 2000), directly estimate dispersal rates (Bradbury et al., 2011), and identify skiped spawning cod remaining on the feeding grounds (Skjæraasen et al., 2012), among other questions. Whether ~80% assignment success is sufficiently high will depend on the specific question, but it does represent a significant increase in power over microsatellites, and, to our knowledge, is among the highest in marine fishes to date.

Increasing assignment capabilities in the marine environment may have implications for the future of Pacific cod fisheries management. Currently, Pacific cod established fishery management units may not reflect suspected stock structure (Spies, 2012). Although the Bering Sea/Aleutian Islands management area was separated into two units beginning in 2015, there is evidence that stocks may be subdivided over smaller spatial scales. For example, cod fisheries in the U.S. Exclusive Economic Zone (EEZ; 3–200 nm offshore) in the Gulf of Alaska Management Unit fall under federal authority, while the State of Alaska manages the fishery within state territorial (0–3 nm) waters. Typically, the Alaska Department of Fish and Game (ADF&G) creates parallel fishing seasons, allowing vessels to fish for cod at the same time as federally controlled fisheries. The significant differentiation between KOD03 and PWS12 found in this study suggests potential isolation of the Prince William Sound population, which is managed by ADF&G and may require independent assessment to annual federal stock assessment reports (Thompson & Palsson, 2013). When management units and associated harvest quotas are not based on realized stock structure, the spatial allocation of fishing may not be proportionate to stock biomass and could result in localized overfishing, particularly if two or more stocks are managed as one (Spies & Punt, 2015). Moreover, disproportionate exploitation of certain stocks may have the potential to destabilize the adaptive resilience of the stock complex as a whole (Hilborn, Quinn, Schindler, & Rogers, 2003).

The success of population assignment tests is not only limited by the power of genetic markers, but also by the availability of baseline samples for assignment. In marine fishes with relatively poorly known and potentially spread‐out spawning locations (Neidetcher et al., 2014) as well as IBD patterns, such population assignment can become very problematic. However, by interpolating allele frequencies in a very defined IBD relationship, SCAT (Wasser et al., 2004) provided some very promising identification of location of origin. To the best of our knowledge, this study is the first to use this method in a low *F*_ST_ marine fish species. Misassignment distances (i.e., the distance between capture location and putative location of birth) in this study (median = 220 km) were greater than generational dispersal from IBD patterns (<100 km; Cunningham et al., 2009). Numerous factors may contribute to this discrepancy. First, there is undoubtedly a statistical error in the estimation of location of origin, although further work is required to quantify that error. A sign of this statistical error may be the tendency of assigning cod to locations at the center of the distribution, both in the Alaskan peninsula/Gulf of Alaska and in the Washington/BC area (Figure 6). In an IBD pattern, misassigments to adjacent locations are expected (and indeed observed with traditional population assignment, Figures S2–S8), but this tendency toward the center of the distribution may have more to do with geographic location of samples and edge effects. Second, SCAT assigns individuals to a population of origin based on their genetic background. As such, progeny of immigrants may have an inflated migration distance that is influenced by their pedigree. Third, although every effort was made to collect spawning fish, samples may have unintentionally included some skip spawners from external populations, thus inflating misassignment and potentially biasing baseline allele frequencies. Skipped spawning has been documented in a wide range of marine species and likely occurs also in Pacific cod (Rideout & Tomkiewicz, 2011).

Despite the challenges of fine‐scale geographic assignment, the results observed here can serve as a starting point for using genetics to investigate movement patterns of Pacific cod and other marine species. Tagging studies (Nichol, Kotwicki, & Zimmermann, 2013; Shimada & Kimura, 1994) have indicated seasonal movements “related to, but not limited to, spawning and feeding” (Rand et al., 2014), but coherent spatiotemporal patterns have not emerged. Although no range‐wide studies have been performed, Shimada and Kimura (1994) estimated that 15%–17% of Pacific cod from the eastern Bering Sea may migrate to the Gulf of Alaska to spawn, and a significant proportion of the stock may undertake large migrations (>100 km; Rand et al., 2014; Shimada & Kimura, 1994). Currently, tagging studies are the standard method of studying movement patterns of Pacific cod, but such studies are expensive in terms of ship time, labor intensive (requires capturing the same fish twice), potentially affected by tagging mortality and atypical behavior of tagged fish, and often limited to few fish being recovered. In contrast, with genetic tools, all individuals in a population are effectively “tagged,” and so all captured individuals can be assigned to a geographic location of origin, resulting in considerably larger sample sizes and more effective coverage of the population. Results from this study suggest that assignment methods may provide an informed approach toward resolving seasonal movement; fish captured during the nonspawning season (April–December) could be assigned geographically using SNP data to putative spawning source populations collected from January to March. This approach may also allow distinction of stationary (i.e., nonmigratory) and migratory ecotypes that are suggested in Pacific cod (Rand et al., 2014) and documented in Atlantic cod (Karlsen et al., 2013).

## 5 CONCLUSIONS

Our study provided unprecedented resolution of the stock structure of Pacific cod along the North American coast. Although previously identified isolation‐by‐distance patterns were confirmed, the higher power of RAD sequencing provided highly significant differentiation among almost all samples. Population structure relevant to management was evident on large (Washington/British Columbia vs. Alaska) as well a small geographic scales (e.g., Prince William Sound vs Kodiak Island), thus providing important information for the definition of management units. Potentially, more importantly, we established the utility of assignment to both population and location of origin and applied that latter approach for the first time in a marine species. SNP‐based genetic stock identification has the potential to provide deeper resolution of population structure and understanding of seasonal migrations in a species where spatial dynamics are not well characterized (Neidetcher et al., 2014). Knowledge of seasonal movement and mixing may provide support for the development and implementation of management practices, such as determination of stock‐specific total allowable catches designed to preserve spatiotemporal stock structure and avoid local depletion. More generally, the application of next‐generation sequencing techniques in combination with sophisticated statistical approaches may provide a significant advance in the management of exploited marine species such as Pacific cod.

## CONFLICT OF INTEREST

None declared.

## DATA ARCHIVE STATEMENT

Sequence data for this study have been deposited at the Short Read Archive of NCBI, accession number SRP137021 (https://www.ncbi.nlm.nih.gov/sra/SRP137021). Scripts and genotype data are available from the Dryad Digital Repository: https://doi.org/10.5061/dryad.402sb71
