# Supplementary material for: Population assignment and local adaptation along an isolation‐by‐distance gradient in Pacific cod (Gadus macrocephalus)
Source: Evol Appl. 2018 May 23;11(8):1448–64. doi: 10.1111/eva.12639 (PMC6100185; doi:10.1111/eva.12639)
Supplement: Supplementary file 1 [file EVA-11-1448-s001.pptx]

## Slide 1
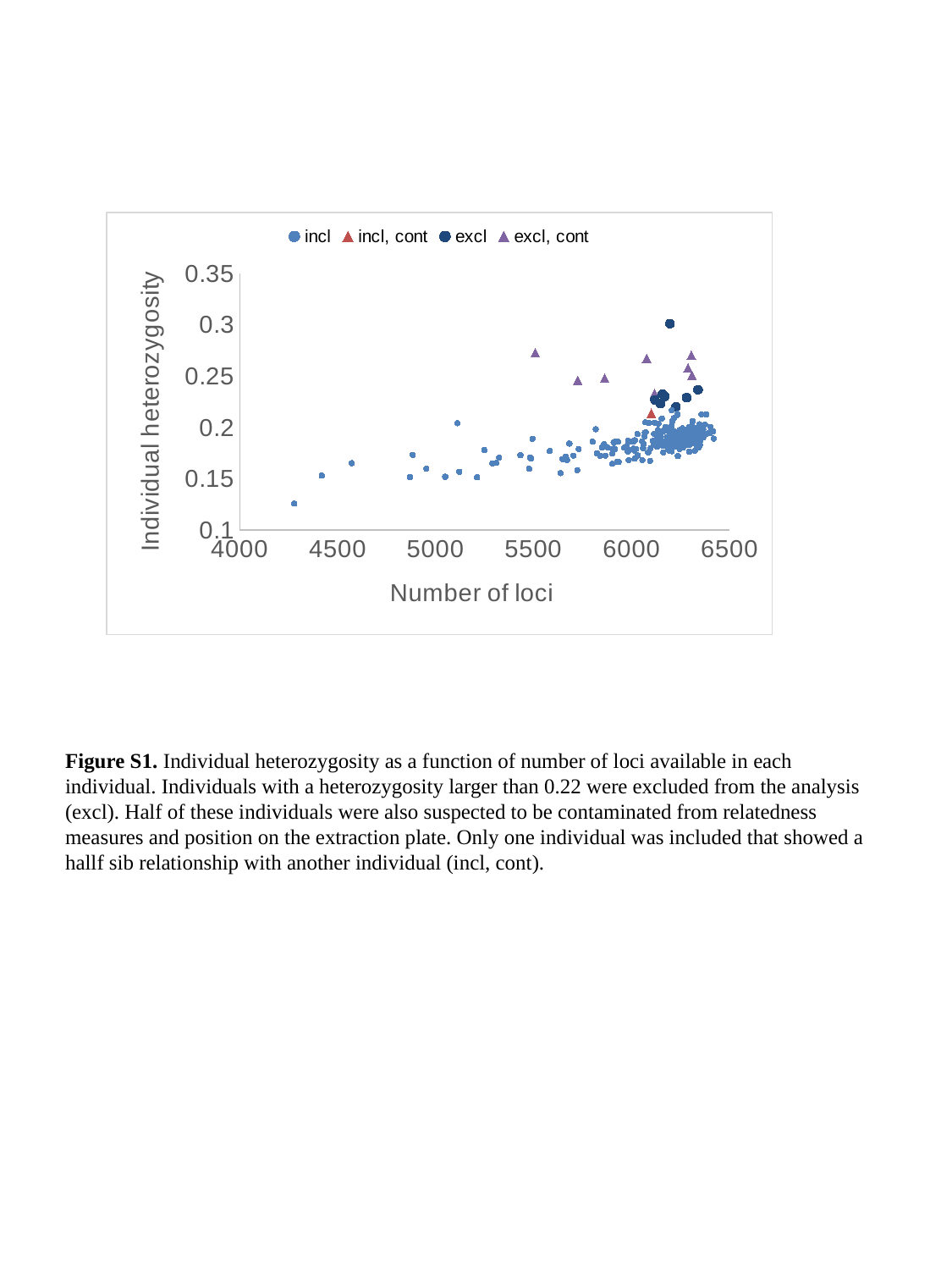

### Chart
| Category | incl | incl, cont | excl | excl, cont |
|---|---|---|---|---|Figure S1. Individual heterozygosity as a function of number of loci available in each individual. Individuals with a heterozygosity larger than 0.22 were excluded from the analysis (excl). Half of these individuals were also suspected to be contaminated from relatedness measures and position on the extraction plate. Only one individual was included that showed a hallf sib relationship with another individual (incl, cont).

## Slide 2
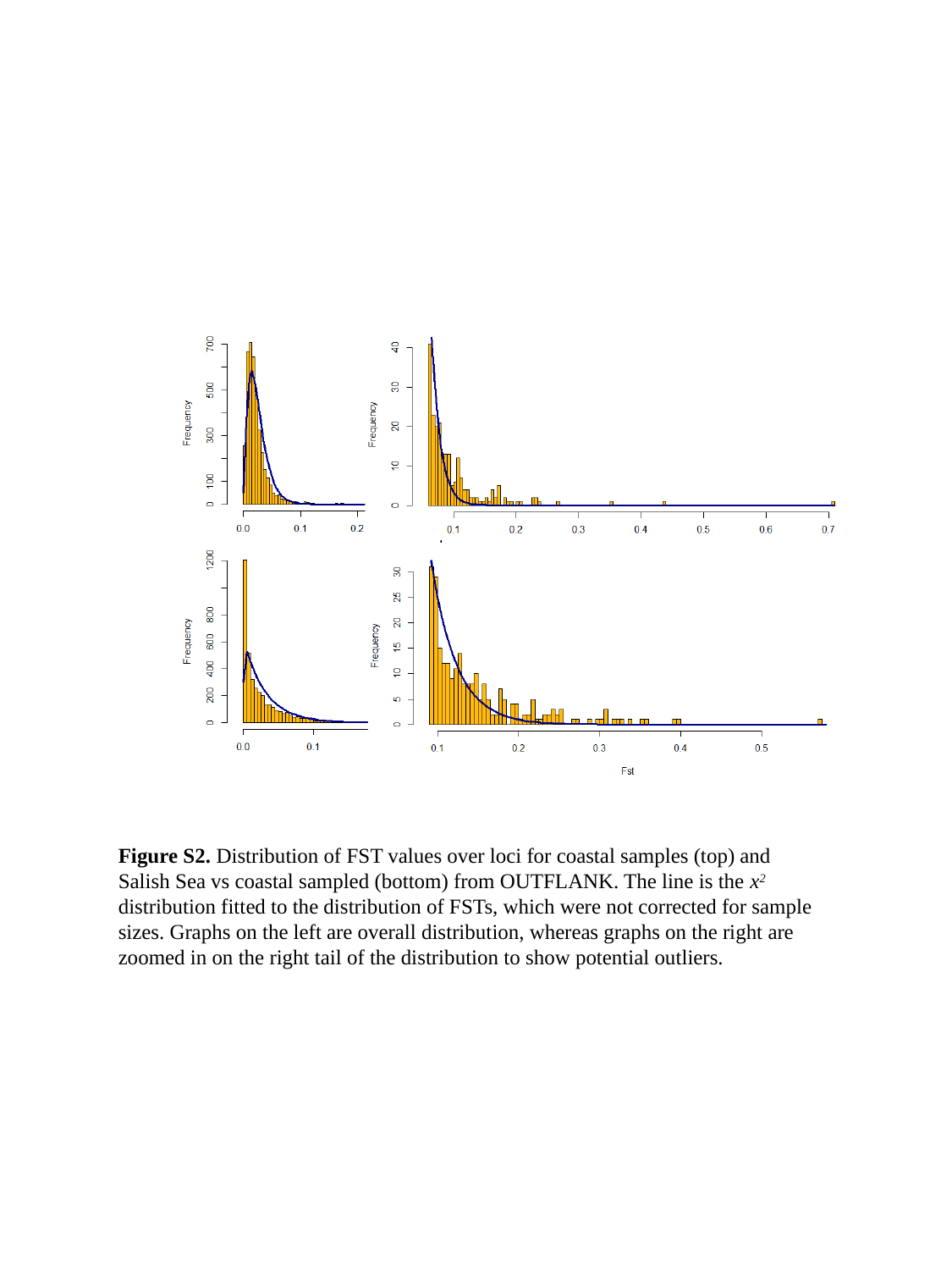

Figure S2. Distribution of FST values over loci for coastal samples (top) and Salish Sea vs coastal sampled (bottom) from OUTFLANK. The line is the x2 distribution fitted to the distribution of FSTs, which were not corrected for sample sizes. Graphs on the left are overall distribution, whereas graphs on the right are zoomed in on the right tail of the distribution to show potential outliers.

## Slide 3
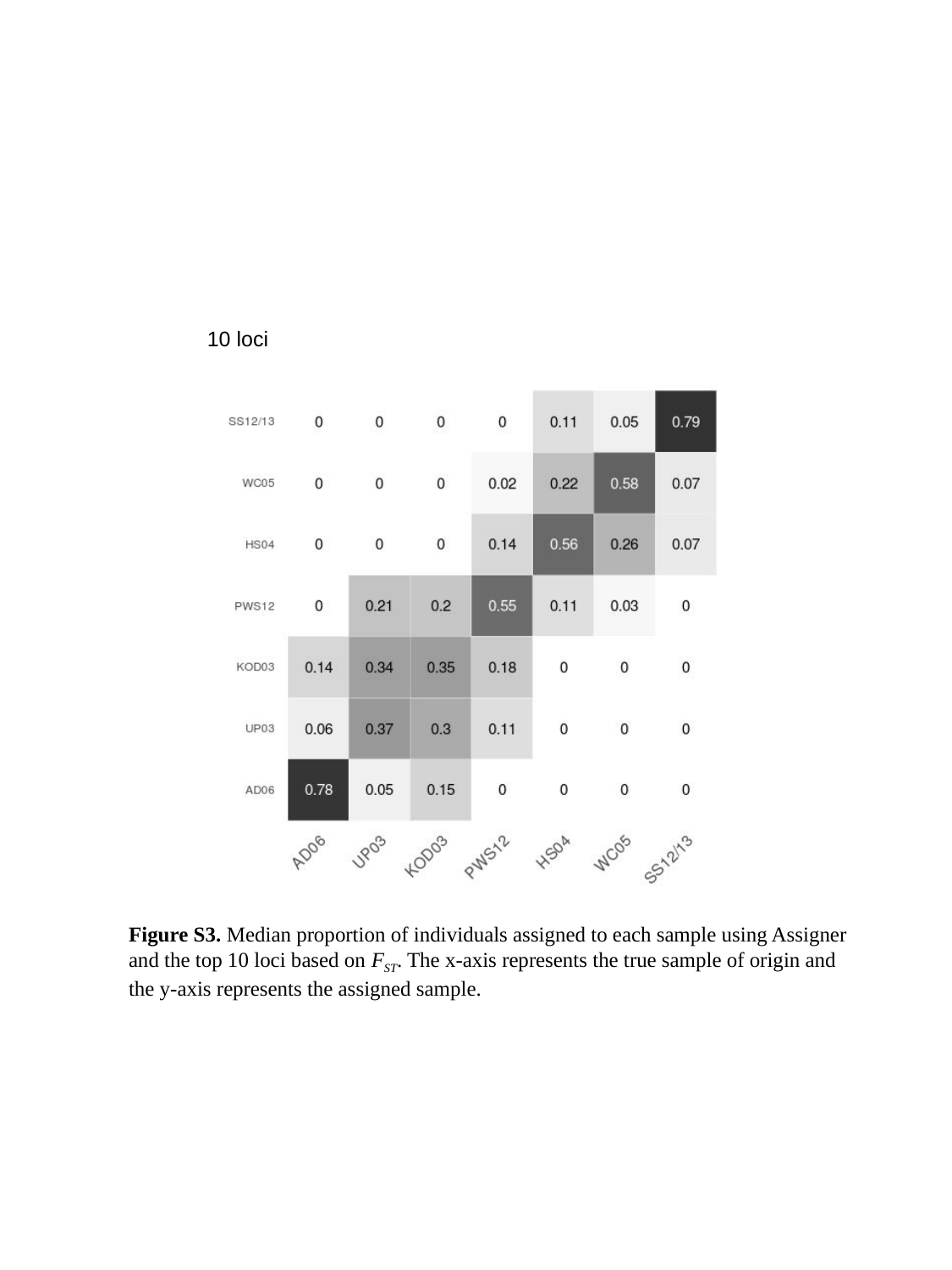

10 loci
Figure S3. Median proportion of individuals assigned to each sample using Assigner and the top 10 loci based on FST. The x-axis represents the true sample of origin and the y-axis represents the assigned sample.

## Slide 4
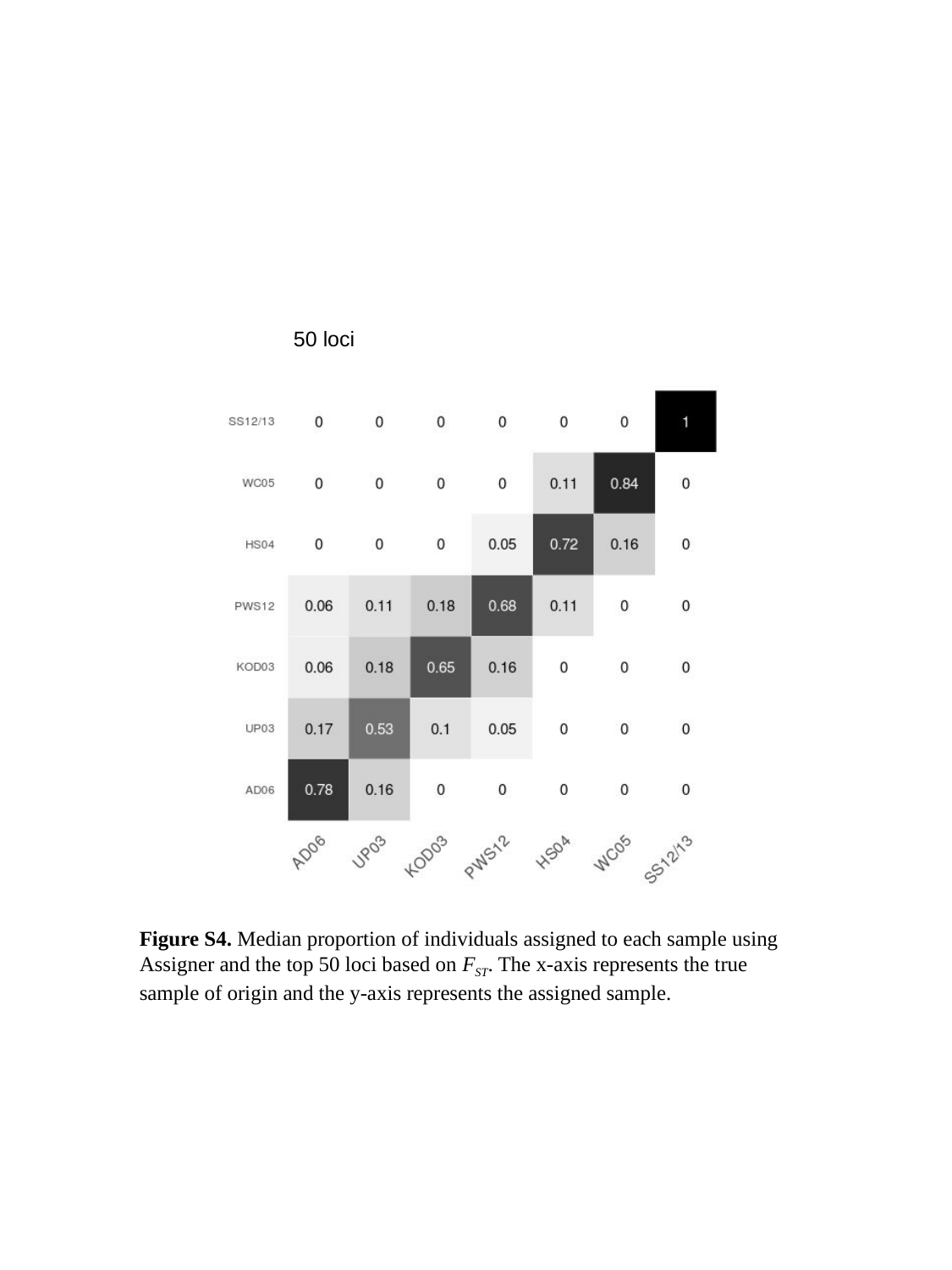

50 loci
Figure S4. Median proportion of individuals assigned to each sample using Assigner and the top 50 loci based on FST. The x-axis represents the true sample of origin and the y-axis represents the assigned sample.

## Slide 5
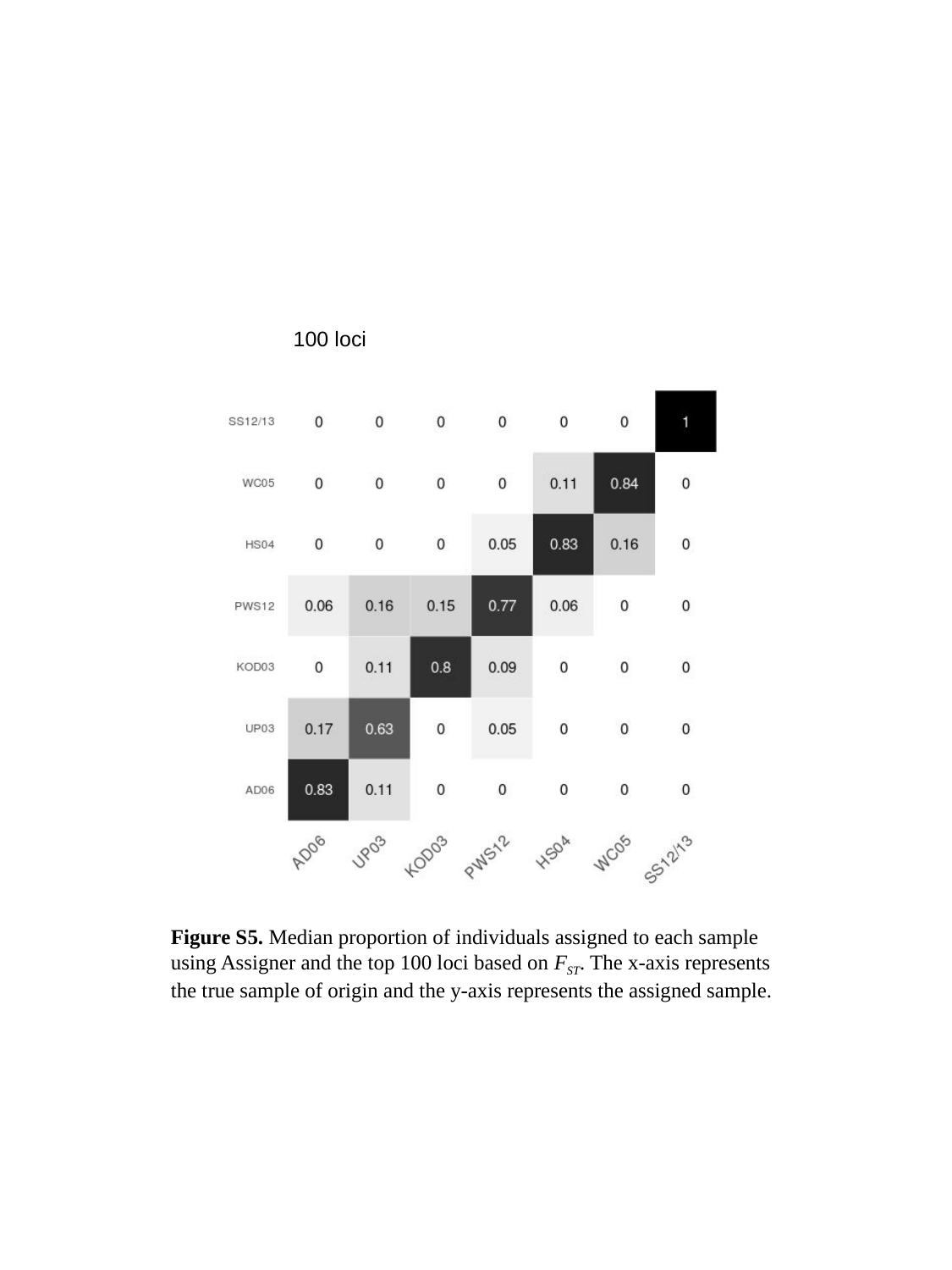

100 loci
Figure S5. Median proportion of individuals assigned to each sample using Assigner and the top 100 loci based on FST. The x-axis represents the true sample of origin and the y-axis represents the assigned sample.

## Slide 6
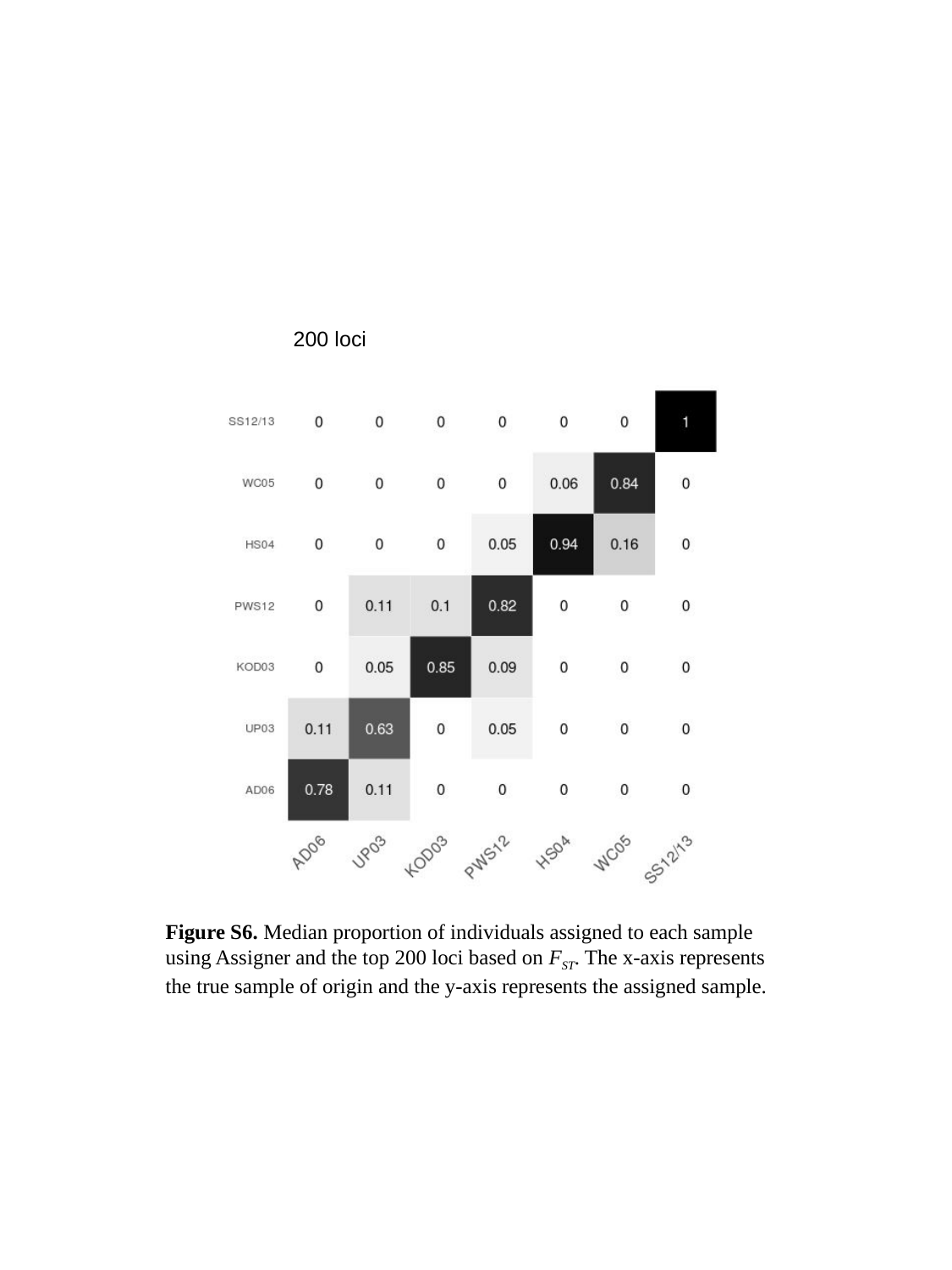

200 loci
Figure S6. Median proportion of individuals assigned to each sample using Assigner and the top 200 loci based on FST. The x-axis represents the true sample of origin and the y-axis represents the assigned sample.

## Slide 7
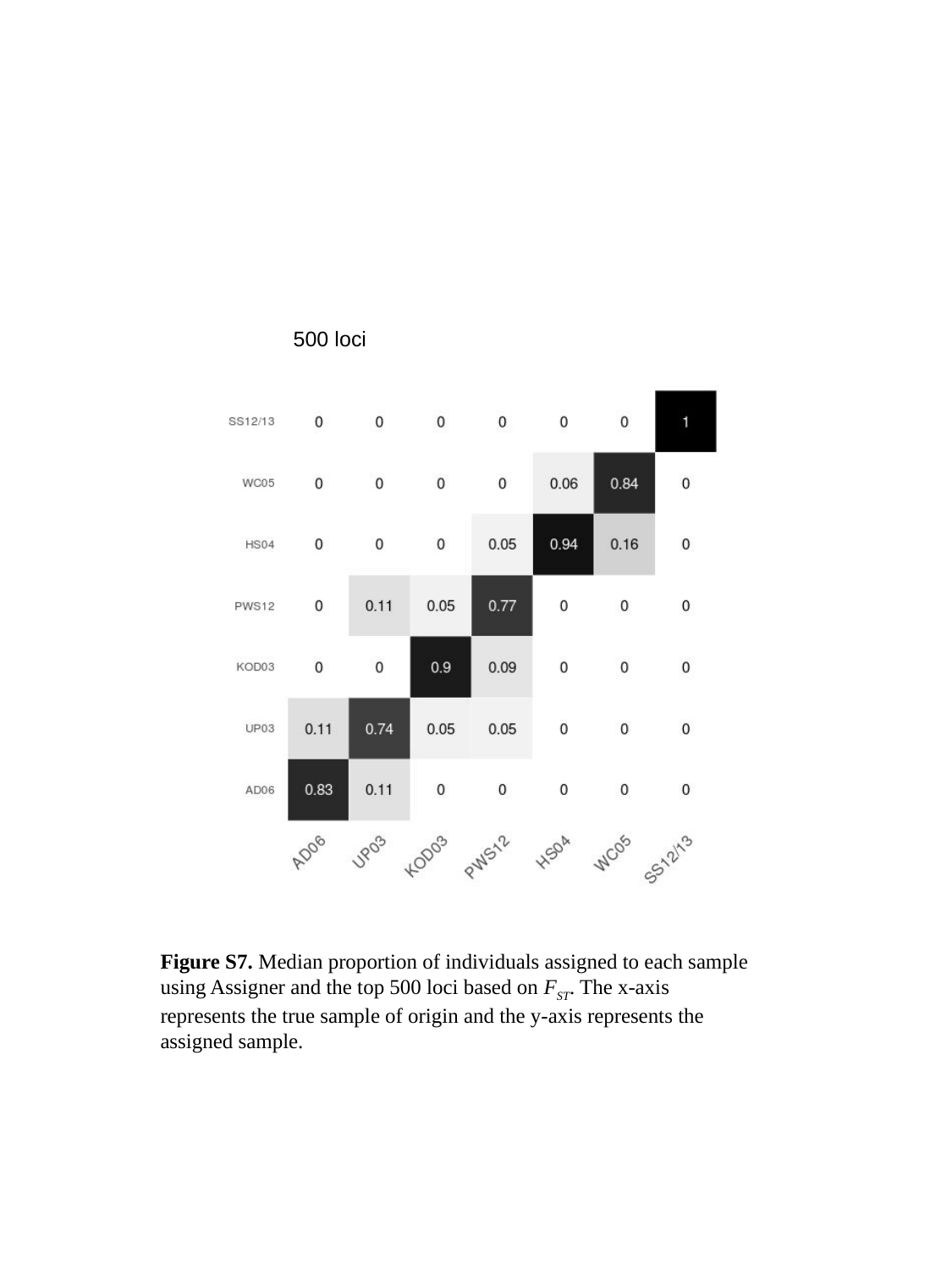

500 loci
Figure S7. Median proportion of individuals assigned to each sample using Assigner and the top 500 loci based on FST. The x-axis represents the true sample of origin and the y-axis represents the assigned sample.

## Slide 8
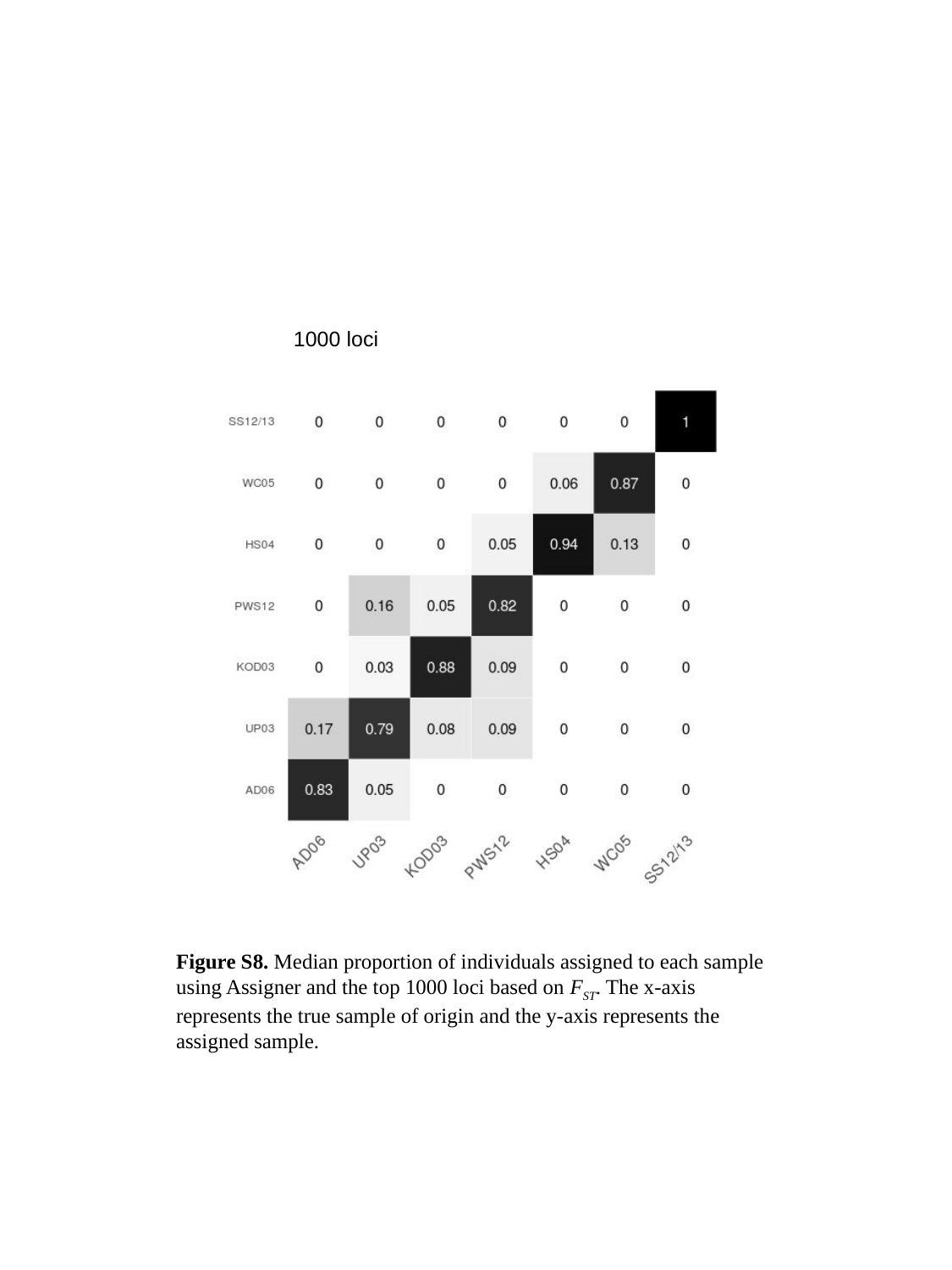

1000 loci
Figure S8. Median proportion of individuals assigned to each sample using Assigner and the top 1000 loci based on FST. The x-axis represents the true sample of origin and the y-axis represents the assigned sample.

## Slide 9
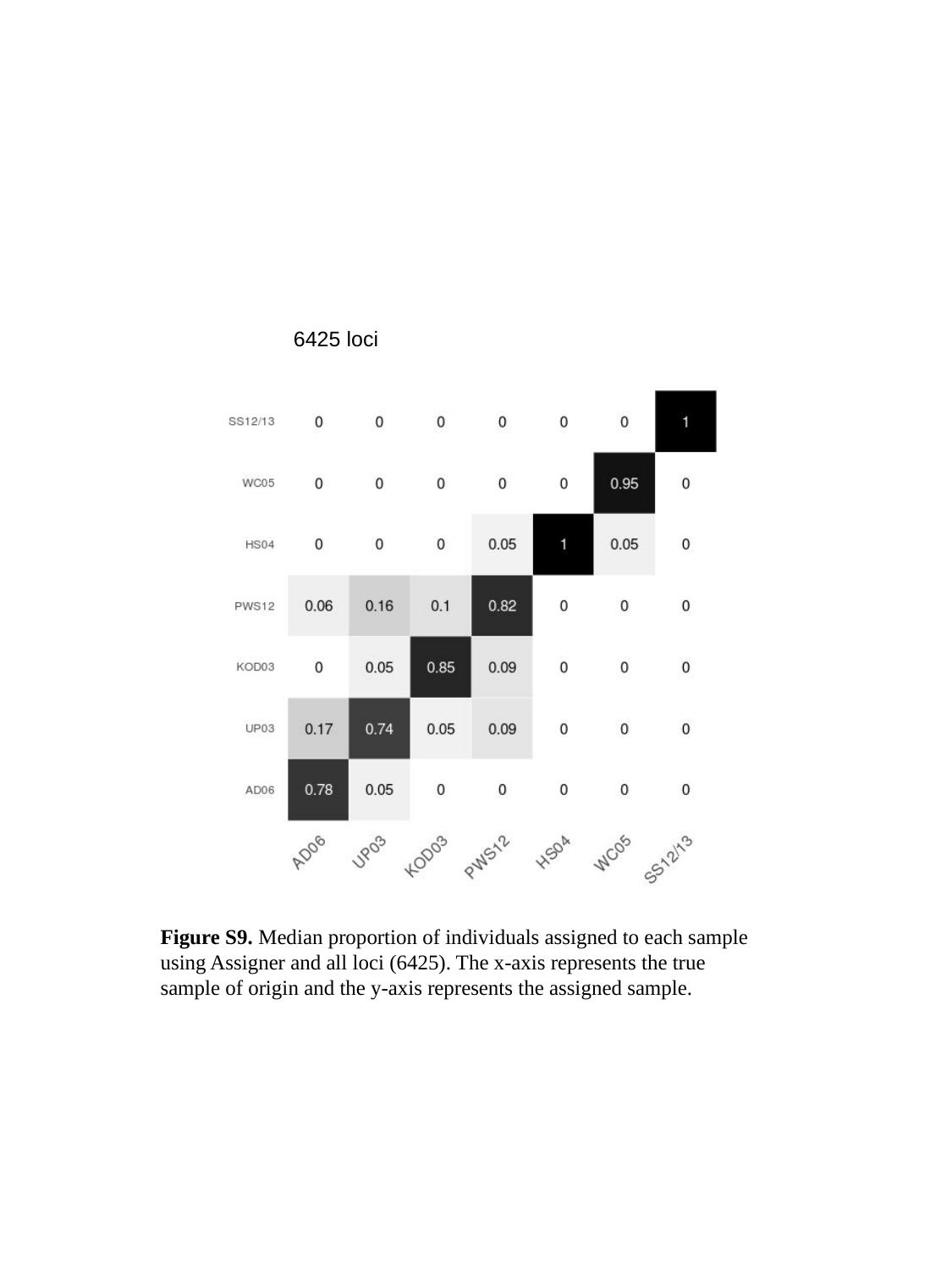

6425 loci
Figure S9. Median proportion of individuals assigned to each sample using Assigner and all loci (6425). The x-axis represents the true sample of origin and the y-axis represents the assigned sample.

## Slide 10
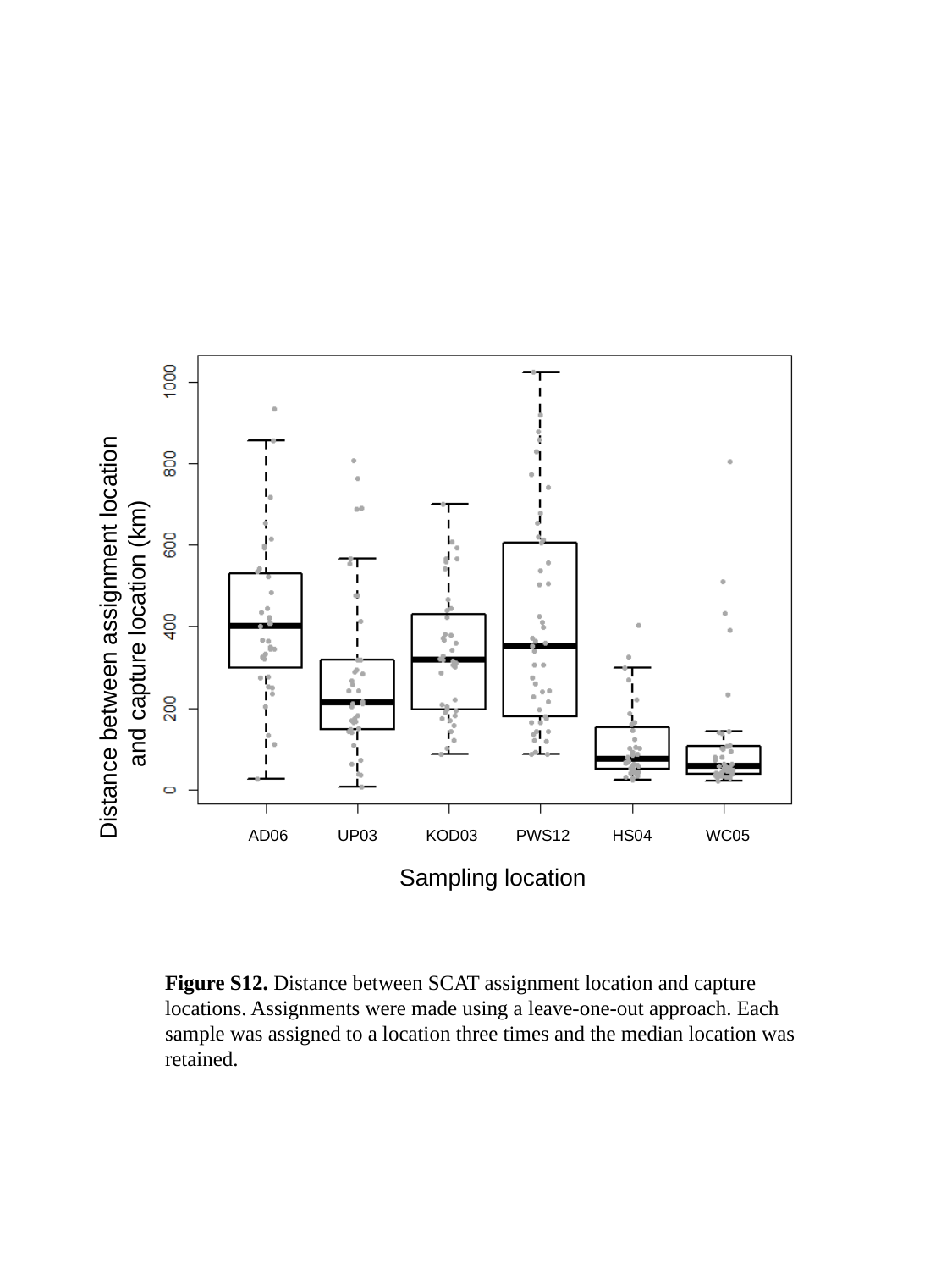

Distance between assignment location
and capture location (km)
AD06
UP03
KOD03
PWS12
HS04
WC05
Sampling location
Figure S12. Distance between SCAT assignment location and capture locations. Assignments were made using a leave-one-out approach. Each sample was assigned to a location three times and the median location was retained.
